# Clinical signs predictive of influenza virus infection in Cameroon

**DOI:** 10.1371/journal.pone.0236267

**Published:** 2020-07-23

**Authors:** Chavely Gwladys Monamele, Cyprien Kengne-Nde, Hermann Landry Munshili Njifon, Mohamadou Ripa Njankouo, Sebastien Kenmoe, Richard Njouom

**Affiliations:** 1 Virology Department, Centre Pasteur of Cameroon, Yaounde, Cameroon; 2 Evaluation and Research Unit, National AIDS Control Committee, Yaounde, Cameroon; Loyola University Health System, UNITED STATES

## Abstract

Influenza virus accounts for majority of respiratory virus infections in Cameroon. According to the World Health Organization (WHO), influenza-like illnesses (ILI) are identified by a measured temperature of ≥38°C and cough, with onset within the past 10 days. Other symptoms could as well be observed however, none of these are specific to influenza alone. This study aimed to determine symptom based predictors of influenza virus infection in Cameroon. Individuals with ILI were recruited from 2009–2018 in sentinel sites of the influenza surveillance system in Cameroon according to the WHO case definition. Individual data collection forms accompanied each respiratory sample and contained clinical data. Samples were analyzed for influenza using the gold standard assay. Two statistical methods were compared to determine the most reliable clinical predictors of influenza virus activity in Cameroon: binomial logistic predictive model and random forest model. Analyses were performed in R version 3.5.2. A total of 11816 participants were recruited, of which, 24.0% were positive for influenza virus. Binomial logistic predictive model revealed that the presence of cough, rhinorrhoea, headache and myalgia are significant predictors of influenza positivity. The prediction model had a sensitivity of 75.6%, specificity of 46.6% and AUC of 66.7%. The random forest model categorized the reported symptoms according to their degree of importance in predicting influenza virus infection. Myalgia had a 2-fold higher value in predicting influenza virus infection compared to any other symptom followed by arthralgia, head ache, rhinorrhoea and sore throat. The model had a OOB error rate of 25.86%. Analysis showed that the random forest model had a better performance over the binomial regression model in predicting influenza infection. Rhinorrhoea, headache and myalgia were symptoms reported by both models as significant predictors of influenza infection in Cameroon. These symptoms could be used by clinicians in their decision to treat patients.

## Introduction

Respiratory infections pose a substantial burden to the healthcare system. Worldwide the burden of lower respiratory tract infection is estimated at 2.3 million deaths per annum [1] meanwhile mortality due to influenza alone accounts for 290 000 to 650 000 deaths [2]. In Cameroon, previous data shows that influenza virus accounts for majority of respiratory virus infections [3]. The case definition of influenza-like illness according to the World Health Organization (WHO) comprises an acute respiratory infection with a measured temperature of ≥38°C and cough, with onset within the past 10 days [4]. Other symptoms could as well be observed in patients with influenza disease including sore throat, chills, malaise, body aches, headache, weakness, myalgia, and coryza [5, 6]. However, none of these symptoms are specific to influenza.

The gold standard assay for diagnosing influenza virus is Reverse Transcriptase Polymerase Chain Reaction (RT-PCR) [7], however few hospitals in low-income settings have the technical platform to perform molecular assays and diagnosis is not routinely performed except within the sentinel hospitals of the influenza surveillance systems. Clinical predictors for influenza have been investigated in several countries [5, 8–12] to help in circumstances where laboratory confirmation is not feasible. These studies reported varying performances based on the study setting and the method used in the analysis.

Due to similar clinical presentations to other infectious agents, presumptive treatment of influenza in hospital settings usually involves the administration of antimalarial and antibacterial drugs which could have as consequence the development of anti-microbial resistance [13]. Meanwhile, previous genomic data has shown that neuraminidase inhibitors are potent antivirals that could be used for influenza therapy in Cameroon [14–17]. Thus, predicting influenza can have important healthcare benefits to the individual as well as to the community. This study aimed to determine symptom based predictors of influenza virus infection in Cameroon using two different statistical methods: binomial logistic predictive model and random forest model.

## Materials and methods

### Description of the influenza surveillance system and recruitment of participants

The Centre Pasteur of Cameroon (CPC) is the National Influenza Centre (NIC) in Cameroon and has implemented influenza sentinel surveillance for more than a decade. This system presently comprise of 16 sites located in seven of the ten administrative regions of Cameroon as previously described [18]. Recruitment of participants was based on the WHO case definition for ILI (Influenza like illness) and SARI (Severe Acute Respiratory Illness). ILI is defined as an acute respiratory infection with measured temperature of ≥38°C and cough, with onset within the past 7 days (10 days since 2016), whereas SARI has an additional requirement of necessitating hospitalization. Influenza surveillance is performed year-round since the implementation of the surveillance system in Cameroon.

### Data collection and analysis

Respiratory samples collected participants who presented with ILI or SARI were sent to the CPC for influenza diagnosis. Individual data collection forms accompanied each respiratory sample and contained clinical data such as: type of respiratory illness (ILI or SARI), clinical signs and anterior or prescribed treatment. At CPC, the QIAamp Viral RNA Mini Kit (Qiagen, Hilden, Germany) was used to extract RNA from clinical samples following the manufacturer’s instructions. Extracts were analyzed for the presence of influenza with the CDC Influenza A/B typing assay, a real time Polymerase Chain Reaction (qPCR), in an ABI Prism 7300 or 7500 thermocycler (Applied Biosystems, Foster City, California, USA). All PCR reactions were performed with Invitrogen SuperScript^™^ III Platinum One-step Quantitative RT-PCR System (ThermoFisher Scientific, Massachusetts, USA) or Ambion AgPath-ID^™^ One-Step RT-PCR Kit (ThermoFisher Scientific, Massachusetts, USA). Samples with threshold cycles (Ct) below 37 were considered positive for influenza.

### Statistical analysis

Two methods were compared to determine the most reliable clinical predictors of influenza virus activity in Cameroon: binomial logistic predictive model and random forest model. For each of the methods we divided the samples into two: a training data set (used to build the models) and a test data set (used to assess the prediction performance of the models). The samples were selected randomly in order to obtain approximately 80% for the training data set and 20% for the test data set. Performance of both models was assessed. The choice of the best binomial logistic predictive model was based on the Akaike Information Criteria (AIC). The area under the curve (AUC) was used to assess the performance of the binomial logistic predictive model while the out-of-bag (OOB) error rate was used to measure the performance of the random forest model. All statistical analyses were performed in R version 3.5.2.

### Ethics statement

Data reported in this study were obtained as part of the influenza surveillance activity which is coordinated by Centre Pasteur of Cameroon, the National Influenza Centre. Individual ethical approval was therefore not required at inclusion. However, individual sample collection proceeded only after acceptance by the person or guardian and after written informed consent was provided. All individual data were anonymized with a code and were entered in a password protected database.

## Results

### Description of the study population

From 2009–2018, a total of 11816 respiratory samples were collected, of which, 2838 (24.0%) were positive for influenza virus by RT-PCR. Amongst these, 38.0% were positive for A(H3N2), 33.2% for influenza B, 20.8% for A(H1N1)pdm09, 6.4% for untyped A, 1.3% for A/B co-infection and 0.1% for the pre-2009 A(H1N1). There was gender equity in the study population with a slightly higher influenza positivity rate in females (24.2%) than males (23.1%). The 0–1 years age group possessed the lowest proportion of influenza cases (16.2%) meanwhile higher proportions was found in the 2–4, 5–14 and 15–49 years age groups at ≥29%. Majority of the population had ILI (86.3%) with an influenza positivity rate of 24.8% compared to 18.7% in SARI cases (Table 1).

**Table 1:** Characteristics of the study population.

	No. tested N (%)	Influenza virus positive N (%)	OR	P-value
**Age group (years)**				
0–1	4884 (41.3)	791 (16.2)	Ref	Ref
2–4	2823 (23.9)	818 (29.0)	2.11	<0.001
5–14	1464 (12.4)	467 (31.9)	2.42	<0.001
15–49	1443 (12.2)	431 (29.9)	2.20	<0.001
50–64	202 (1.7)	53 (26.2)	1.84	<0.001
≥ 65	101 (0.9)	27 (26.7)	1.88	0.005
No data	899 (7.6)	248 (27.6)		
**Gender**				
Male	5459 (46.2)	1261 (23.1)	0.94	0.191
Female	5392 (45.6)	1303 (24.2)	Ref	Ref
No data	965 (8.2)	271 (28.1)		
**Type of illness**				
ILI	10193 (86.3)	2531 (24.8)	1.43	<0.001
SARI	1547 (13.1)	290 (18.7)	Ref	Ref
No data	76 (0.6)	14 (18.4)		
**Total**	**11816**	**2835 (24.0)**		

Table 2 shows the proportion of symptoms reported in the study population with respect to influenza status. Majority of the persons presented with cough (92.1%), rhinorrhoea (88.0%) and asthenia (71.1%). Amongst the 11/14 symptoms that were significantly associated with influenza positivity, cough, rhinorrhoea, asthenia, head-ache, myalgia and arthralgia were more frequent in persons positive for influenza (P-value < 0.05). Meanwhile; vomiting, shortness of breath, diarrhoea and skin rash were more frequent in persons negative for influenza.

**Table 2:** Proportion of symptoms with respect to influenza status.

Symptoms	Proportion n/N (%)			P-value
	Overall	Influenza negative	Influenza positive	
Cough	10006/10864 (92.1)	7509/8263 (90.9)	2497/2601 (96.0)	< 0.001*
Rhinorrhoea	9203/10453 (88.0)	6872/7930 (86.7)	2331/2523 (92.4)	< 0.001*
Asthenia	6210/8738 (71.1)	4575/6596 (69.4)	1635/2142 (76.3)	< 0.001*
Headache	3135/6286 (49.9)	2167/4623 (46.9)	968/1663 (58.2)	< 0.001*
Sore throat	2215/6400 (34.6)	1628/4726 (34.4)	587/1674 (35.1)	0.654
Vomitting	2411/8680 (27.8)	1913/6606 (29.0)	498/2074 (24.0)	< 0.001*
Arthralgia	1528/5521 (27.7)	1052/4095 (25.7)	476/1426 (33.4)	< 0.001*
Noisy breath	2103/8473 (24.8)	1746/6448 (27.1)	357/2025 (17.6)	< 0.001*
Myalgia	1301/5583 (23.3)	902/4148 (21.7)	399/1435 (27.8)	< 0.001*
Shortness of breath	1862/8290 (22.5)	1546/6295 (24.6)	316/1995 (15.8)	< 0.001*
Diarrhoea	1690/8616 (19.6)	1428/6591 (21.7)	262/2025 (12.9)	< 0.001*
Conjonctivitis	1608/8357 (19.2)	1214/6353 (19.1)	394/2004 (19.7)	0.581
Ear pain	917/6144 (14.9)	690/4571 (15.1)	227/1573 (14.4)	0.539
Skin rash	707/8024 (8.8)	596/6096 (9.8)	111/1928 (5.8)	< 0.001*

Regarding the type of illness, the following symptoms were more frequently recorded among individuals positive for influenza who presented with ILI: cough, rhinorrhoea, asthenia, head ache, arthralgia and myalgia. On the other hand, SARI cases had cough as the only symptom that was significantly associated with influenza positivity. Whereas, in both ILI and SARI cases, several symptoms were more frequently noted in persons negative for influenza including: vomiting, noisy breath, shortness of breath and diarrhoea (Table 3).

**Table 3:** Proportion of symptoms with respect to type of illness.

Symptoms	ILI n/N (%)		P-value	SARI n/N (%)		P-value
	Influenza Neg	Influenza Pos		Influenza Neg	Influenza Pos	
Cough	6422/7019 (91.5)	2237/2332 (95.9)	< 0.001*	1039/1192 (87.2)	249/256 (97.3)	< 0.001*
Rhinorrhoea	5883/6715 (87.6)	2102/2254 (93.3)	< 0.001*	944/1164 (81.1)	220/256 (85.9)	0.073
Asthenia	3770/5524 (68.2)	1450/1906 (76.1)	< 0.001*	772/1023 (75.5)	174/223 (78.0)	0.438
Headache	1886/3932 (48.0)	900/1493 (60.3)	< 0.001*	267/650 (41.1)	61/160 (38.1)	0.530
Sore throat	1366/3944 (34.6)	542/1492 (36.3)	0.252	252/743 (33.9)	41/170 (24.1)	0.014*
Vomitting	1478/5528 (26.7)	420/1838 (22.9)	0.001*	425/1030 (41.2)	75/223 (33.6)	0.035*
Arthralgia	885/3455 (25.6)	437/1263 (34.6)	< 0.001*	161/601 (26.8)	38/154 (24.7)	0.682
Noisy breath	1334/5390 (24.7)	287/1794 (16.0)	< 0.001*	404/1010 (40.0)	68/218 (31.2)	0.017*
Myalgia	769/3495 (22.0)	363/1269 (28.6)	< 0.001*	128/612 (20.9)	32/157 (20.4)	1.000
Shortness of breath	1174/5263 (22.3)	260/1759 (14.8)	< 0.001*	362/984 (36.8)	53/223 (23.8)	< 0.001*
Diarrhoea	1085/5515 (19.7)	219/1794 (12.2)	< 0.001*	339/1028 (33.0)	42/218 (19.3)	< 0.001*
Conjonctivitis	993/5291 (18.8)	362/1764 (20.5)	0.108	210/1015 (20.7)	30/227 (13.2)	0.009*
Ear pain	615/3861 (15.9)	212/1391 (15.2)	0.577	70/669 (10.5)	15/171 (8.8)	0.572
Skin rash	509/5102 (10.0)	93/1699 (5.5)	< 0.001*	84/947 (8.9)	17/216 (7.9)	0.690

Neg: Negative; Pos: Positive

With respect to age, we categorized our study population into three groups: 0–4 years (young children), 5–14 years (old children), ≥15 years (adults). Cough, rhinorrhea, and head ache were the three symptoms found to be significantly associated to influenza positivity in all age groups. Additional symptoms could be noted amongst the young children (asthenia) and adult population (asthenia, arthralgia). Table 4 describes the frequency of symptoms with respect to influenza status and age group.

**Table 4:** Proportion of symptoms with respect to age groups.

Symptoms	0–4 years n/N (%)		P-value	5–14 years n/N (%)		P-value	≥15 years n/N (%)		P-value
	Inf Neg	Inf Pos		Inf Neg	Inf Pos		Inf Neg	Inf Pos	
Cough	5528/5981 (92.4)	1533/1583 (96.8)	< 0.001*	855/970 (88.1)	442/462 (95.7)	< 0.001*	1021/1200 (85.1)	474/508 (93.3)	< 0.001*
Rhinorrhoea	5232/5770 (90.7)	1465/1537 (95.3)	< 0.001*	715/897 (79.7)	394/445 (88.5)	< 0.001*	835/1165 (71.7)	429/496 (86.5)	< 0.001*
Asthenia	3114/4777 (65.2)	907/1284 (70.6)	< 0.001*	553/728 (76.0)	317/396 (80.1)	0.135	858/1028 (83.5)	399/445 (89.7)	0.002*
Headache	681/2732 (24.9)	250/812 (30.8)	0.001*	530/763 (69.5)	288/377 (76.4)	0.014*	928/1079 (86.0)	419/455 (92.1)	0.001*
Sore throat	582/2961 (19.7)	144/862 (16.7)	0.054	348/691 (50.4)	163/346 (47.1)	0.356	679/1028 (66.1)	269/449 (59.9)	0.025*
Vomitting	1575/5005 (31.5)	356/1313 (27.1)	0.002*	180/674 (26.7)	105/366 (28.7)	0.513	133/865 (15.4)	32/376 (8.5)	0.001*
Arthralgia	240/2529 (9.5)	74/697 (10.6)	0.386	198/578 (34.3)	102/303 (33.7)	0.881	605/952 (63.6)	295/413 (71.4)	0.005*
Noisy breath	1450/4847 (29.9)	255/1270 (20.1)	< 0.001*	142/674 (21.1)	44/353 (12.5)	0.001*	132/863 (15.3)	55/385 (14.3)	0.669
Myalgia	182/2578 (7.1)	63/718 (8.8)	0.127	174/591 (29.4)	81/302 (26.8)	0.434	538/941 (57.2)	248/401 (61.8)	0.116
Shortness of breath	1115/4698 (23.7)	179/1249 (14.3)	< 0.001*	131/655 (20.0)	41/342 (12.0)	0.001*	276/880 (31.4)	94/388 (24.2)	0.011*
Diarrhoea	1212/4992 (24.3)	208/1281 (16.2)	< 0.001*	105/670 (15.7)	27/350 (7.7)	< 0.001*	295/413 (71.4)	27/378 (7.1)	0.038*
Conjonctivitis	846/4716 (17.9)	232/1260 (18.4)	0.711	139/670 (20.7)	62/341 (18.2)	0.360	220/910 (24.2)	97/387 (25.1)	0.725
Ear pain	295/3000 (9.8)	72/844 (8.5)	0.289	127/624 (20.4)	42/320 (13.1)	0.007*	264/907 (29.1)	112/397 (28.2)	0.791
Skin rash	503/4587 (11.0)	78/1207 (6.5)	< 0.001*	58/623 (9.3)	22/341 (6.5)	0.143	30/830 (3.6)	9/364 (2.5)	0.378

Inf: Influenza; Neg: Negative; Pos: Positive

### Symptom-based prediction of influenza virus infection

Binomial logistic predictive model revealed that the presence of cough, rhinorrhoea, headache and myalgia are significant predictors of influenza positivity with an AIC of 3769.5. Cough and rhinorrhea had twice higher odds of being observed in influenza positive cases than in negative cases. Likewise, shortness of breath and diarrhea had lower odds of occurring in influenza positive cases (P-value < 0.05; Table 5).

**Table 5:** Binomial logistic prediction model.

Symptoms	Estimate	OR	P-value
Cough	0.860	2.4	< 0.001*
Rhinorrhoea	0.811	2.3	< 0.001*
Asthenia	0.155	1.2	0.094
Headache	0.382	1.5	< 0.001*
Sore throat	0.161	1.2	0.117
Vomitting	-0.116	0.9	0.293
Arthralgia	0.170	1.2	0.139
Noisy breath	-0.177	0.8	0.169
Myalgia	0.291	1.3	0.019*
Shortness of breath	-0.529	0.6	< 0.001*
Diarrhoea	-0.660	0.5	< 0.001*
Conjonctivitis	-0.222	0.8	0.071
Ear pain	-0.265	0.7	0.054
Skin rash	-0.352	0.7	0.076

**Fig 1:**
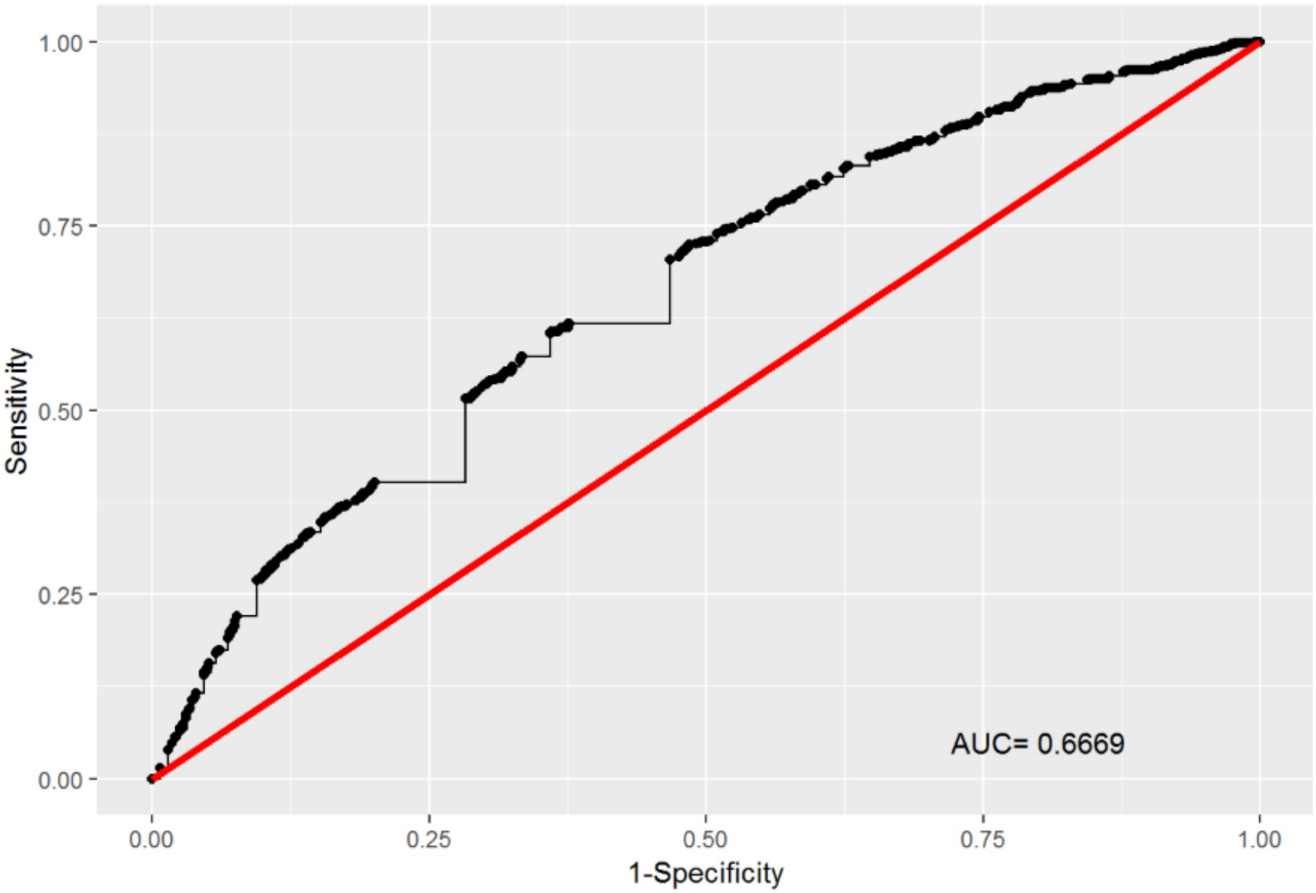
Performance of the binomial logistic predictive model.

The prediction model had a sensitivity (Se) of 75.6%, specificity (Sp) of 46.6% and AUC of 66.7%, as indicated by the ROC curve (Fig 1).

**Fig 2:**
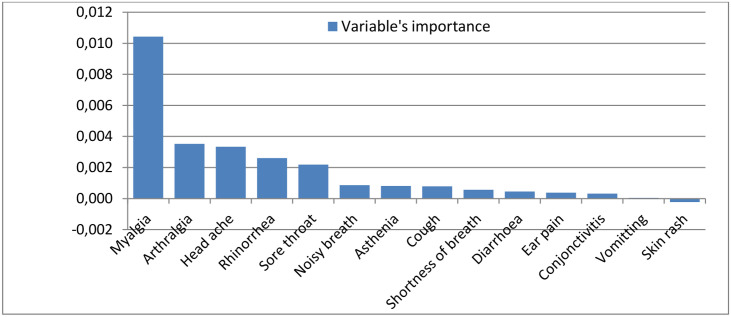
Variable’s importance for clinical prediction of influenza.

The random forest model categorized the reported symptoms according to their degree of importance in predicting influenza virus infection. Fig 2 shows a graphical representation of the results obtained with the random forest model. Myalgia had a 2-fold higher value in predicting influenza virus infection compared to any other symptom followed by arthralgia, head ache, rhinorrhoea and sore throat. Meanwhile, the presence of skin rash and vomiting were less likely to indicate an influenza virus infection. The model had a OOB error rate of 25.86%.

## Discussion

This study evaluated two methods for predicting influenza virus infection in Cameroon based on symptoms reported by each case-patient. Comparison of both methods showed that the random forest model had a better performance compared to the binomial regression model. Random forest had an OOB error rate of 25.86% indicating an accuracy of about 74%. Meanwhile, binomial regression model had a moderate sensitivity (75.6%) and a poor specificity (46.6%). Rhinorrhoea, headache and myalgia were symptoms reported by both models as significant predictors of influenza infection. In the random forest model, arthralgia and sorethroat were as well important predictors of influenza positivity; meanwhile in the binomial regression model cough was the additional clinical predictor of importance. Results obtained with the binomial regression model is comparable to reports by Casalegno et al. who noted that cough, fever, headache, weakness, myalgia and coryza were associated with an increased risk of influenza infection [6]. Similarly, Shah et al. in 2015 showed that cough, fever, rhinorrhea and myalgia were clinical predictors of the presence of influenza virus [8] with moderate to good sensitivity and poor specificity. In our study, fever was not evaluated as a distinct symptom since the case definition for influenza-like illness already included fever.

In the other hand, results obtained with the random forest model is comparable to reports by Anderson et al in 2018 who found that fever, cough, sore throat, chills, malaise and body ache were indicative of influenza infection. However, chills and malaise are not part of the symptoms that are recorded amongst influenza suspect cases in Cameroon and could therefore not be evaluated. Previous studies noted that case definition for influenza infection was age-dependent, especially in children where the presence of fever or rhinorrhoea had an increased sensitivity in indicating influenza infection [8, 9]. The higher representation of children in our study population could be a reason why rhinorrhea was a significant predictor of influenza infection. Varying results observed in other studies can be attributed to the different methods and approaches used or due to differences in the target population. Indeed, several authors instead evaluated the performance of different case definition [6, 8, 19, 20] as opposed to evaluating the performance of each symptom as presented in this study. Also, several other methods have been used by other authors notably the Classification and Regression Tree-CART [5], logistic regression [8, 9] and generalized estimating equations-GEE model [6]. These differences render comparison accross studies difficult.

Negative predictors for influenza infection could be assessed with the binomial logistic regression model. Shortness of breath and diarrhea were the two symptoms reported as less indicative of the presence of influenza infection. These results corroborate with previous reports who found that individuals who were influenza-positive were less likely to have diarrhea, difficulty breathing, dyspnea, wheezing or lung findings on physical exam than non-influenza ILI cases [5]. However, due to the less likelihood of seeking for negative predictors in individuals with ILI, we did not emphasize much on these negative predictors.

A main limitation to this study was the fact that some patient file did not have complete information on the symptoms presented biasing the overall results obtained.

## Conclusions

Comparison of two methods for determining the best clinical predictors of influenza virus infection showed that the random forest model had a better performance compared to the binomial regression model. Rhinorrhoea, headache and myalgia were symptoms reported by both models as significant predictors of influenza infection in Cameroon. These symptoms could be used by clinicians in their decision to treat patients.
